# Occurrence of Antimicrobial Resistance in Indicator Bacteria and *Campylobacter* spp. Isolated from Commercial Raw-Meat-Based Food for Dogs and Cats in Belgium

**DOI:** 10.3390/antibiotics15030282

**Published:** 2026-03-10

**Authors:** Junjia He, Ilias Chantziaras, Cristina Garcia-Graells, Moniek Ringenier, Suzanne Dewulf, Filip Boyen, Jeroen Dewulf, Cécile Boland

**Affiliations:** 1 Veterinary Epidemiology Unit, Faculty of Veterinary Medicine, Ghent University, Salisburylaan 133, 9820 Merelbeke, Belgium; 2 Veterinary Bacteriology Service, Department of Infectious Diseases in Animals, Sciensano, Juliette Wytsmanstraat 14, 1050 Brussels, Belgium; 3 Foodborne Pathogens Service, Department of Human Infectious Diseases, Sciensano, Juliette Wytsmanstraat 14, 1050 Brussels, Belgium; 4 Department of Pathobiology, Pharmacology and Zoological Medicine, Faculty of Veterinary Medicine, Ghent University, 9820 Merelbeke, Belgium

**Keywords:** raw meat-based diets, antimicrobial resistance, multidrug resistance, companion animals, food safety, *Escherichia coli*, *Enterococcus faecium*, *Enterococcus faecalis*, *Campylobacter*

## Abstract

**Background:** Raw-meat-based diets (RMBDs) for companion animals have gained popularity but may serve as vehicles for antimicrobial-resistant (AMR) bacteria, posing risks to animal and public health. This study investigated the occurrence and risk factors of AMR in indicator bacteria (*Escherichia coli*, *Enterococcus faecalis*, *Enterococcus faecium*) and *Campylobacter* spp. from commercial RMBD products. **Methods:** In 2023, 50 RMBD samples were collected in Belgium, representing 21 brands from five countries. After both selective and non-selective isolation and MALDI-TOF identification, antimicrobial susceptibility testing of the isolates was performed using broth microdilution. **Results:** From non-selective media, *E. coli* was found in 45 samples (90.0%), *E. faecalis* in 31 samples (62.0%), *E. faecium* in 23 samples (46.0%), and *Campylobacter* spp. in 3 samples (6.0%). Among these, one *E. faecalis* strain with acquired resistance to vancomycin and daptomycin was isolated. Multidrug resistance (MDR) was identified in 17 isolates from 15 samples (30.0%), including 14 MDR *E. coli*, 1 MDR *E. faecalis*, and 2 MDR *E. faecium*. From selective media, presumptive ESBL/AmpC-producing *E. coli* were detected in 17 samples (34.0%), and 5 *E. faecium* from linezolid-supplemented media were confirmed by the broth microdilution method. Samples from Belgian origin showed significantly higher *E. faecium* prevalence (76.5%) compared to Dutch samples (21.4%) (OR = 11.9, *p* < 0.001). Minor livestock sources were associated with increased MDR risk (OR = 5.52, *p* = 0.016). **Conclusions:** Commercial RMBDs in Belgium exhibit widespread bacterial contamination with concerning AMR patterns. These findings highlight the need for improved production standards in the RMBD industry and the need to raise awareness in pet owners.

## 1. Introduction

The emergence and spread of antimicrobial-resistant (AMR) bacteria pose a significant threat to animal and public health. The World Health Organization (WHO) has identified AMR as one of the top ten global public health threats facing humanity [1]. For the monitoring of antimicrobial resistance, the AMR occurrence in *Escherichia coli* (*E. coli*) is recognized as an indicator of the level of AMR in Gram-negative bacteria, while *Enterococcus faecalis* (*E. faecalis*) and *Enterococcus faecium* (*E. faecium*) are used as indicators of AMR in Gram-positive bacteria [2]. The AMR level of *Campylobacter* spp. (in particular *C. coli* and *C. jejuni*) is also monitored at the European level, as it is an important foodborne zoonotic agent [2]. These indicator and pathogenic bacteria have the ability to acquire and disseminate resistance genes, which could lead to difficult-to-treat infections in both humans and animals [3,4].

Raw-meat-based diets (RMBDs) for companion animals, such as dogs and cats, have gained popularity among pet owners [5]. These diets are often perceived as more natural and healthier alternatives to conventional pet foods. However, the raw nature of these products presents multiple risks, including that they may serve as vehicles for importing AMR pathogens into household environments. As a matter of fact, a landmark study from the Netherlands found that 80% of commercial raw pet foods contained extended-spectrum β-lactamase-producing *E. coli* [6] and a cluster of four Shiga toxin-producing *Escherichia coli* (STEC) O157:H7 infections in the UK, including one fatal case of haemolytic uraemic syndrome, was epidemiologically linked to the handling of raw pet food [7]. More recently, a *Salmonella* outbreak in Canada (37 illnesses, 8 hospitalizations) was associated with raw pet food [8]. In addition, a previous study has shown that up to 70% of raw-meat products did not meet European Union hygiene standards, particularly regarding acceptable levels of *Enterobacteriaceae*, *E. coli*, and *Salmonella* contamination [9]. Unlike heat-treated pet foods, raw-meat products provide optimal conditions for bacterial survival and growth, as pathogenic bacteria are not eliminated during processing, which can then be transmitted to humans through direct contact with pets, handling of pet food, or environmental contamination [10,11,12]. Previous research has indicated that *E. coli* and *Campylobacter jejuni* are commonly found in RMBDs [13], while *E. faecalis* and *E. faecium* have also been detected in such products [14]. Moreover, studies have identified the presence of AMR bacteria in RMBDs, not only Enterobacteriaceae [9] but also *Campylobacter* spp. [15] and *Enterococcus* species [16,17], suggesting a potential transmission pathway to pets and their human contacts. According to 2022 data, over 50% of European families raise at least one pet, with dogs and cats representing the majority of pets [18]. This raises concerns about cross-species transmission and the broader implications for antimicrobial resistance, leading to higher morbidity, mortality, and healthcare costs [19,20]. Therefore, this study aims to investigate the occurrence of AMR in indicator bacteria (*E. coli*, *E. faecium*, and *E. faecalis*) and *Campylobacter* spp., as a major foodborne pathogen with zoonotic potential, isolated from commercial raw-meat-based pet food products in Belgium, and the potential associated risk factors. Risk factors investigated included production location, animal source, and tissue type, as these factors reflect differences in regulatory frameworks, antimicrobial usage patterns, and biosecurity levels across production systems that may influence AMR occurrence [21,22,23,24].

## 2. Results

### 2.1. Sample Collection and Properties

A total of 703 products were identified based on a search in physical and online pet shops. Out of these, 148 contained insects or seafood as primary ingredients and were excluded, leaving 555 eligible products. From these 555 eligible products, a total of 50 samples were selected through proportional random sampling of products purchased from 13 stores, including 8 shops (35 samples) with frozen-chain delivery service and the remaining 15 samples of products from 5 stores where the samples were picked up by the first author.

**Figure 1:**
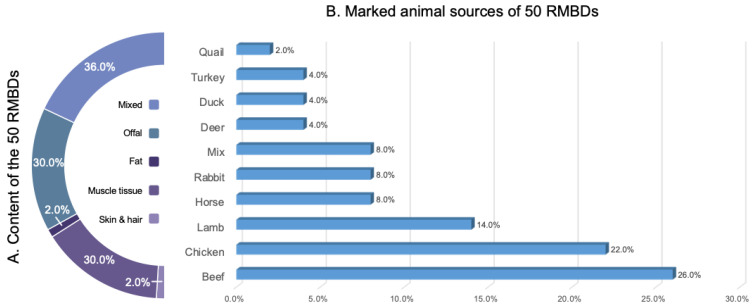
Types of products and animal sources of 50 RMBDs.

According to the information marked on the product, these 50 samples represented 21 different brands and were produced in five countries: Belgium (17/50, 34.0%), the Netherlands (28/50, 56.0%), France (1/50, 2.0%), Germany (3/50, 6.0%), and Spain (1/50, 2.0%). The marked animal sources included quail (1/50 sample, 2.0%), turkey (2/50, 4.0%), duck (2/50, 4.0%), deer (2/50, 4.0%), rabbit (4/50, 8.0%), horse (4/50, 8.0%), lamb (7/50, 14.0%), chicken (11/50, 22.0%), beef (13/50, 26.0%), and mixed species (4/50, 8.0%) (Figure 1). In terms of content, the products included different parts of the animals, such as muscle tissues (15/50, 30.0%), offal (heart, liver, kidney, intestines, stomach and other edible organ tissues) (15/50, 30.0%), fat (1/50, 2.0%), and skin with hair (1/50, 2.0%), some products consisted of mixtures of different tissues (18/50, 36.0%) (Figure 1).

### 2.2. Detection of Bacteria

Out of the 50 collected RMBD samples, *E. coli* was isolated from 45 samples (90.0%), *E. faecalis* from 31 samples (62.0%), *E. faecium* from 23 samples (46.0%), and *Campylobacter* spp. from 3 samples (6.0%). In one sample, all four targeted bacterial species were isolated, whereas in 11 samples (22.0%), three bacterial species were isolated; in 28 samples (56.0%), two species were isolated; in 9 samples (18.0%), only one species was isolated; and in only one sample, none of the bacterial species were isolated.

#### Selective Isolation of Resistant Strains

Additionally, 17 *E. coli* isolates were obtained from MacConkey agar plates supplemented with 1 mg/L cefotaxime. Three *E. faecalis* and five *E. faecium* strains were isolated from Slanetz–Bartley agar plates supplemented with 4 mg/L linezolid and were further tested by the broth microdilution method to confirm them as non-wild-type for linezolid.

### 2.3. Antimicrobial Susceptibility Results

Results of the antimicrobial susceptibility testing (minimum inhibitory concentrations (MICs) distributions) are summarized in Table 1, Table 2 and Table 3 for *E. coli*, *E. faecalis*, and *E. faecium* isolated from non-antibiotic-supplemented agar, respectively.

Among the 45 *E. coli* isolates obtained from RMBDs, 37.8% (17/45) exhibited acquired resistance to ampicillin, and 31.1% (14/45) showed acquired resistance to tetracycline. Acquired resistance was also observed against sulfamethoxazole (26.7%, 12/45), cefotaxime and ceftazidime (both 24.4%, 11/45), and trimethoprim and ciprofloxacin (both 15.6%, 7/45), while lower percentages were found for nalidixic acid (11.1%, 5/45), chloramphenicol (6.7%, 3/45), colistin (4.4%, 2/45) and gentamicin (2.2%, 1/45). All *E. coli* isolates obtained from non-selective supplemented agar belonged to the wild-type population for amikacin, azithromycin, meropenem and tigecycline (Table 1).

*E. faecalis* is intrinsically resistant to quinupristin/dalfopristin, with 90.3% (28/31) showing MIC values above the EFSA ECOFF set for data reporting purposes [2]. For tetracycline, 51.6% (16/31) exhibited acquired resistance, whereas for erythromycin this was 25.8% (8/31). Acquired resistance was also found for ampicillin, daptomycin and vancomycin (all 3.2%, 1/31). All these 31 *E. faecalis* isolates belonged to the wild-type population for chloramphenicol, ciprofloxacin, gentamicin, linezolid, teicoplanin, and tigecycline (Table 2).

Out of the 23 *E. faecium isolates,* 60.9% (14/23) showed acquired resistance to quinupristin/dalfopristin, 30.4% (7/23) to tetracycline and 13.0% (3/23) to erythromycin. Lower percentages of resistance were found for ampicillin, ciprofloxacin (both 8.7%, 2/23), chloramphenicol and linezolid (both 4.3%, 1/23). All these 23 *E. faecium* isolates belonged to the wild-type population for daptomycin, gentamicin, teicoplanin, vancomycin, and tigecycline (Table 3).

Three *Campylobacter* spp. isolates were obtained from the 50 samples: two *Campylobacter jejuni* and one *Campylobacter coli.* The two *C. jejuni* showed the same resistance profiles—both showed acquired resistance to ertapenem and ciprofloxacin, and belonged to the wild-type population for other tested antibiotics (chloramphenicol, erythromycin, gentamicin, and tetracycline). The only *C. coli* exhibited acquired resistance to ertapenem and was wild-type for ciprofloxacin, chloramphenicol, erythromycin, gentamicin, and tetracycline.

Out of the 45 *E. coli* strains isolated from non-selective media, 14 of them exhibited acquired multidrug resistance (MDR), more specifically, acquired resistance to 3–7 antibiotic classes (antimicrobial categories considered for MDR classes were summarized in Supplementary Material Table S1). Among the 31 *E. faecalis* and 23 *E. faecium* isolates, 3 strains exhibited acquired MDR: 1 *E. faecalis* strain and 2 *E. faecium* strains. In total, 17 MDR isolates were identified from the non-selective media in this study, distributed across 15 samples. “EC07”, “EFS1” and “EFM2” were isolated from the same sample. Their resistance profiles are listed in Table 4.

Regarding the 17 *E. coli* strains isolated from MacConkey agar plates supplemented with 1 mg/L cefotaxime, the MICs detailed in the Supplementary Material Table S2 indicate presumptive ESBL/AmpC phenotypes for all these isolates.

Three *E. faecalis* strains initially grown on Slanetz–Bartley agar plates supplemented with 4 mg/L linezolid could not be confirmed as non-wild-type for linezolid by the broth microdilution method with both 24 h and 48 h incubation (MIC ≤ 4 mg/L).

For *E. faecium*, after 24 h of incubation of the broth microdilution plates, three out of the five *E. faecium* grown on Slanetz–Bartley supplemented with linezolid exhibited an MIC above the linezolid ECOFF (Supplementary Table S3), while after 48 h, all five *E. faecium* isolates were above the ECOFF for linezolid (>4 mg/L). For chloramphenicol, after 24 h of incubation, two out of the five *E. faecium* strains were above the ECOFF, while after 48 h, all but one strain were above the chloramphenicol ECOFF (>32 mg/L).

### 2.4. Risk Factors

Risk factor analysis was conducted to study factors influencing the prevalence of *E. coli*, *E. faecalis*, and *E. faecium,* and the detection of MDR. *Campylobacter* spp. could not be included in the risk factor analysis due to there being insufficient positive samples.

For this, the production location was categorized as Belgium (BE), Netherlands (NL), and others, to ensure sufficient observations in each category. For animal source classification, this study categorized products into three groups based on production systems and biosecurity control levels: Conventional Livestock (beef, lamb, chicken, turkey), representing primary livestock species with standardized production systems and regulatory oversight; Minor Livestock (duck, quail, horse, rabbit) comprised smaller production systems in the countries of origin; and Wild-Sourced Animals (deer, wild animals) included products from natural environments with minimal biosecurity control and no standardized health monitoring. The contingency table and the results of the univariate risk factors analysis are presented in Table 5.

For *E. coli*, production location was a significant risk factor. As no other variables were statistically associated with the prevalence of *E. coli,* no multivariable models were made for *E. coli*.

For *E. faecium*, production location was identified as a significant protective factor; an attempted multivariable model was performed but could not be reliably fitted due to quasi-complete separation in the data when including other variables, so the reported association is based on the univariable analysis in Table 5.

Multivariable logistic regression was attempted for “MDR samples”, including production location (forced entry due to prior hypotheses) and animal source categories as covariates; the multivariable outcomes were not reliably fitted due to quasi-complete separation in the data categorical predictors containing small cell counts. Therefore, the reported associations are based primarily on univariable analysis in Table 5.

Multivariate logistic regression analysis was performed for the *E. faecalis* strains presented, including production location (forced entry), conventional livestocks and wild-sourced animal origin as covariates. After adjusting for other variables, both of the animal origins showed strong protective trends against *E. faecalis* presence, though these did not reach statistical significance (Conventional livestock: adjusted OR[aOR] = 0.1214, 95% CI: 0.014–1.068, *p* = 0.057; Wild Animal: aOR = 0.053, 95% CI: 0.003–1.071, *p* = 0.055). In contrast, the production location showed no significant association with *E. faecalis* present risk (*p* = 0.345).

## 3. Discussion

### 3.1. The Prevalence of Indicator Bacteria and the Occurrence of AMR Patterns

The high isolation rates of indicator bacteria from RMBDs in this study align with previous research but exceed some reported rates in comparable studies. The near-universal presence of *E. coli* (90.0%) suggests widespread contamination, consistent with the inherent risks of unprocessed animal products. This rate was slightly higher than the one observed from another study (86%) in Dutch commercial raw-meat diets [6]. Interestingly, our findings align closely with a study from Italy (98.2%) [25], although no products from that country were included in our study. The persistently high recovery rates across studies in different countries suggest that *E. coli* contamination is an intrinsic characteristic of raw pet food products rather than a region-specific quality issue.

The detection of presumptive ESBL/AmpC-producing *E. coli* in 17 samples (34%, 17/50) represents a significant finding that warrants careful consideration within the broader context of antimicrobial resistance surveillance. This prevalence rate substantially exceeds baseline levels reported in European food production systems, where ESBL/AmpC-producing *E. coli* detection rates from caecal or meat samples averaged 14.3% (338/2370) in the 2021 EU surveillance study [26]. Similarly, the prevalence of ESBL/AmpC *E. coli* in fresh meat from Portugal was 17.1% (109/638) [27], and German meat products (primarily from conventional livestock sources) showed ESBL/AmpC-producing *E. coli* prevalence of 21.2% (304/1435) [28]. However, our findings align more closely with resistance patterns observed in companion animals themselves. Studies from Portugal and the UK reported ESBL/AmpC-positive Enterobacteriaceae prevalence rates of 55.8% (24/43) and 36.4% (8/22) respectively in pets [29]. While these findings suggest a potential association between RMBD contamination and companion animal carriage of resistant bacteria, further research is needed to establish causal relationships and determine the relative contribution of different transmission pathways.

The prevalence of enterococci (*E. faecalis* 62.0%, *E. faecium* 46.0%) observed in this study highlights potential microbial risks associated with raw-pet-food consumption. The overall enterococcal prevalence aligns with the 54% reported in Portuguese pet foods across various product types [16]. Notably, when considering only raw frozen products in their study, a 100% enterococci contamination rate was observed (*n* = 14). Additionally, preliminary screening on 4 mg/L linezolid medium obtained five *E. faecium* strains from different RMBDs (10%, *n* = 5/50) was also worrisome.

The relatively low prevalence of *Campylobacter* spp. (6.0%) in this study was unexpected, given previous reports of *C. jejuni* in 22% of commercial raw pet foods in New Zealand [15] and its common occurrence in raw meat intended for human consumption, where prevalence rates can exceed 30% in poultry products [30,31,32]. This discrepancy may relate to manufacturing and storage processes, as, for example, freezing can significantly reduce *Campylobacter* spp. survival [33]. Indeed, in the study in New Zealand, raw pet food was a freshly purchased, non-frozen product, while in the current study, all RMBD samples were frozen products, which likely contributed to the lower detection rates compared to studies examining fresh or refrigerated products. However, this finding is consistent with some studies that detected no *Campylobacter* in commercial raw pet foods [34,35]. This represents a limitation of our study design, as frozen storage may have reduced the viability of *Campylobacter* and potentially underestimated its true prevalence in fresh RMBD products; it may also indicate that frozen RMBDs pose less risk in this regard. Consistent with the known epidemiology of these pathogens, two out of the three *Campylobacter* spp. isolates originated from products containing poultry meat (both were *Campylobacter jejuni)*, while the *Campylobacter coli* isolate originated from a product containing lamb.

The widespread bacterial contamination across different animal sources and product types in our study demonstrates that bacterial presence is not confined to specific meat categories but rather represents an inherent characteristic of raw pet food products, regardless of product composition. This finding aligns with previous research showing no significant differences in bacterial contamination rates across different meat types in raw pet diets [36], suggesting that contamination occurs at multiple points throughout the production and distribution chain rather than being source-specific.

### 3.2. Antimicrobial Resistance Patterns Observed

The antimicrobial resistance patterns observed in our isolates raise substantial concerns about RMBDs as potential vehicles of AMR bacteria.

High acquired-resistance rates were observed among *E. coli* isolates to several antibiotics, particularly ampicillin (37.8%), tetracycline (31.1%), sulfamethoxazole (26.7%), and third-generation cephalosporins (cefotaxime 24.4%, ceftazidime 24.4%). Resistance to third-generation cephalosporins is an indication of potential ESBL/AmpC activity, as these enzymes are known to hydrolyze these β-lactam antibiotics while remaining susceptible to β-lactamase inhibitors [37].

In addition, the observed prevalence rate of presumptive ESBL/AmpC-producing *E. coli* in RMBDs (34%, 17/50) in our study, based on the selective isolation, aligns with high contamination levels consistently reported in European RMBD investigations. Swiss commercial raw pet foods showed 62.7% AMR bacteria prevalence, with the majority resistant to third-generation cephalosporins due to ESBL production [9]. Dutch RMBD studies documented ESBL-producing *E. coli* in 80% (28/35) of products [6]. In Sweden, ESC-resistant *E. coli* carrying pAmpC (*bla*_CMY-2) was detected in 23% (9/39) of raw food diets containing poultry [38]. Similarly, American studies reported ESBL-producing Enterobacteriaceae in 42% (10/24) of raw diets [39], demonstrating that high ESBL prevalence in RMBDs is not limited to European production systems. This consistently high prevalence across different countries and manufacturers suggests that ESBL contamination represents an inherent characteristic of raw-pet-food production rather than isolated quality control failures. By comparison, conventional meat products intended for human consumption show variable ESBL rates (4–75% depending on meat type and processing), indicating that the absence of heat-treatment and potential concentration effects during RMBD processing may contribute to elevated resistance burdens in raw pet foods [21,22,23]. Also noteworthy, among the 17 presumptive ESBL/AmpC producing *E. coli* strains isolated through the selective isolation in our study, nearly half (47.1%) showed NWT for ciprofloxacin. This rate of ciprofloxacin resistance among presumptive ESBL/AmpC *E. coli* aligns with the rate observed among such isolates from livestock and conventional meat in Europe (48.7%) [26].

Among the 45 *E. coli* isolates analyzed, 31.1% (*n* = 14) exhibited multidrug resistance. Notably, one strain (EC14 in Table 4) demonstrated acquired resistance to multiple classes of critically important antimicrobials, including to third-generation cephalosporins (ceftazidime), fluoroquinolones (ciprofloxacin), and polymyxins (colistin). The MDR occurrence (31.1%) observed in our study is lower than MDR rates reported in Portuguese studies examining raw pet meat products, which found 71% [40] and 91% [27] MDR rates among *E. coli* isolates. Interestingly, a study of dogs consuming RMBDs versus non-raw diets found a significant difference: 24.6% vs. 3.9% MDR *E. coli*, respectively, in their fecal sample [41]. While these observations do not establish direct causality, it suggests that RMBDs may serve as reservoirs for multidrug-resistant bacteria within the broader antimicrobial resistance ecosystem of the One Health framework [41].

Colistin resistance in 4.4% of *E. coli* isolates is also a matter of concern given colistin’s status as a last-resort antibiotic. This finding aligns with global reports of colistin resistance in pet-associated bacteria across multiple countries [9,42], including direct detection in raw-meat-based diets for pets [40].

High resistance rates were observed among enterococci isolates, particularly to erythromycin (25.8% in *E. faecalis*), tetracycline (51.6% in *E. faecalis* and 30.4% in *E. faecium*), and quinupristin/dalfopristin (60.9% in *E. faecium*; intrinsic resistance in *E. faecalis*). Resistance to these antimicrobial classes is commonly observed in isolates from food-producing animals in Belgium [43].

The multidrug resistance occurrence in *Enterococcus* spp. varies considerably among studies. Our study identified MDR rates of 3.2% in *E. faecalis* and 8.7% in *E. faecium*, contrasting with European reports exceeding 30% for both species in similar food matrices [16,17], yet slightly surpassing US pet food (1.7%; [44]. The observed linezolid resistance rates (*E. faecalis* 0.0%, *E. faecium* 4.3%) were substantially lower than those reported in Portuguese industrial raw dog food (23%; [16]). In the preliminary screening stage, the occurrence of *E. faecium* grown on linezolid-supplemented media was 10% (5/50 RMBDs), confirming the enhanced detection observed with such selective isolation. Notably, for the five *E. faecium* strains grown on media supplemented with linezolid, the results of MICs after 48 h of incubation compared to 24 h of incubation for linezolid and chloramphenicol are in agreement with observations from a previous study [45]. Based on these observations, all five of these *E. faecium* strains could be considered as non-wild-type for linezolid if the 48 h of incubation is used for final classification.

Another finding is the *E. faecalis* isolate with acquired resistance to vancomycin and daptomycin, two antibiotics categorized as to be “avoided” in veterinary medicine according to the European medicine agency classification [46].

### 3.3. Risk Factors

An interesting finding of this study is the association between production location and bacterial contamination patterns, particularly for *E. faecium*. Samples produced in Belgium demonstrated a higher prevalence of *E. faecium* (76.5%) compared to Dutch samples (21.4%). These differences may be due to production practices, regulatory oversight, or supply chain management between countries. Regulatory frameworks governing raw-pet-food production vary across European Union member states, with some countries implementing more stringent microbiological criteria and hazard analysis critical-control point requirements than others [24,47]. Additionally, differences in antimicrobial usage patterns in livestock production between countries could influence the occurrence of resistant bacteria in the food chain, subsequently affecting raw pet food products derived from these sources [48,49,50]. However, no statistical difference in the detection of MDR indicator bacteria was observed when comparing different production locations.

Products containing skin and hair components showed a lower prevalence of *E. faecium*. However, these findings are constrained by incomplete product labelling information and the fact that most products contained multiple tissue types rather than single components. The risk factor analysis could only determine whether products contained specific components, not their relative proportions or processing methods. Additionally, the small sample sizes in some categories (*n* = 6 for skin and hair products) limit the statistical power. Therefore, future studies with detailed compositional analysis and larger sample sizes are needed to validate these preliminary findings.

Analysis of animal source categories revealed only one association between production systems and bacterial contamination patterns. Minor livestock sources were associated with increased multidrug resistance risk. It is difficult to provide a clear explanation for this finding.

The high occurrence of AMR bacteria in commercial RMBDs may pose significant public health risks through multiple transmission pathways. Pet owners may face direct exposure during food handling, preparation, and serving processes, with contaminated hands, utensils, and surfaces serving as potential vectors for bacterial transmission [23]. Additional risks may arise from indirect contact through contaminated feeding equipment, pet saliva, and household surfaces. These findings underscore the need for enhanced consumer education regarding safe handling practices for raw pet foods, including proper hand hygiene, surface disinfection, and separation from human food preparation areas [49]. The integration of RMBD safety into broader One Health surveillance frameworks is essential for monitoring and mitigating AMR transmission risks at the human–animal interface [20].

### 3.4. Study Limitations and Future Research

Several limitations should be acknowledged when interpreting these findings. The relatively small sample size of 50 products may limit generalizability and statistical power for detecting associations. Risk factor analysis was constrained by sample size, particularly for variables with small cell counts. On top of that, given the relatively small sample size and the considerable heterogeneity across brands, batches, and retail sources, the number of samples per cluster was generally too small for reliable variance estimation at the cluster level. Broad categorization of production locations due to limited numbers may have obscured country-specific patterns. The cross-sectional design provides only a snapshot of contamination patterns and cannot establish causal relationships. A significant limitation relates to sample handling and potential contamination during the supply chain. For products obtained through online retailers with delivery services (35/50 samples), contamination during merchant storage and cold-chain delivery could not be controlled or assessed. Similarly, original product packaging integrity and its role in preventing or facilitating contamination was not systematically evaluated. Furthermore, the study relied on product labelling for categorizing animal sources and production locations, which may not accurately reflect actual product composition. Lastly, frozen storage of the products investigated in this study may have reduced the viability of *Campylobacter* and potentially underestimated its true prevalence in fresh RMBD products.

## 4. Materials and Methods

### 4.1. Sampling Methods

RMBD products were collected between May and August 2023 at various retail sources in Belgium, including physical pet supply stores or authorized online retailers that provide delivery services within Belgium. All available RMBD products were identified through information provided by the merchants and basic product details. Only products consisting exclusively of raw meat were included; products containing processed ingredients or preservation additive were excluded. All products that fulfilled the selection criteria were categorized by labelled animal source (bovine, porcine, poultry, and other livestock) to ensure representation across different animal species. To focus on terrestrial-animal-derived raw-meat products, products containing insects or seafood as primary ingredients were excluded. Following this, the remaining eligible products were stratified by the marked animal sources, and a proportional random selection was performed. Subsequently, the selected products were purchased. For purchases made at physical pet supply stores, samples were collected in sterile containers and transported to the laboratory under refrigerated conditions (2–8 °C) within 24 h of collection. For purchases made through online retailers, merchants provided cold-chain delivery to the researcher’s private address, where samples were stored in sterile containers and subsequently transported under refrigeration (2–8 °C) to the laboratory within 24 h. For each eligible product, comprehensive metadata were documented, including brand name, manufacturer details, production date and batch number.

### 4.2. Bacterial Isolation and Identification

Upon arrival at the laboratory, each sample was assigned a unique identifier code, thawed at room temperature (20–25 °C) in a separate homogenizing bag and subsequently homogenized using a laboratory blender (Homogenizer Laboratory Blender, Thermo Scientific, Oxoid™, Basingstoke, UK) for 2 min. A 25 g portion of the sample was aseptically transferred to 225 mL of Buffered Peptone Water (BPW, Biorad, Lokeren, Belgium) and, for incubation, two aliquots were prepared from each sample, one for the isolation of *E. coli*, *E. faecalis*, *E. faecium*, and the other for the isolation of Campylobacter spp.

#### 4.2.1. *E. coli* Isolation

BPW homogenous solution was incubated for 18–22 h at 37 °C. Following the incubation, 10 µL of the enriched solution was streaked onto MacConkey agar (Thermo Scientific™ Oxoid™, Basingstoke, UK) plates using a sterile inoculating loop and incubated aerobically for 18–20 h at 37 °C. Colonies displaying typical *E. coli* morphology (pink to rosy colonies, 2–3 mm diameter) were selected for further purification. Suspected colonies were first streaked onto Tryptone Bile X-glucuronide (TBX, Merck, Darmstadt, Germany) agar to confirm β-glucuronidase activity (blue-green colonies), followed by subculture on Nutrient Agar (Thermo Scientific™ Oxoid™, Basingstoke, UK) to obtain pure colonies for identification. Both plates were incubated aerobically at 37 °C for 18–20 h and 16–24 h, respectively. Bacterial identification was performed using Matrix-Assisted Laser Desorption/Ionization–Time of Flight (MALDI-TOF) mass spectrometry (Bruker MALDI Biotyper, Bruker Daltonics, Bremen, Germany) with the MBT Compass Library (version 2023, encompassing 4320 species across 712 microorganism genera), with a score value ≥ 2.0 considered as reliable species identification.

#### 4.2.2. *E. faecalis* and *E. faecium* Isolation

BPW homogenous solution was incubated for 18–22 h at 37 °C. Following the incubation, 10 µL of the enriched solution was streaked onto Slanetz–Bartley agar (Thermo Scientific™ Oxoid™, Basingstoke, UK) plates using a sterile inoculating loop and incubated aerobically at 42 °C for 44–48 h. Colonies displaying typical enterococcal morphology (red, maroon or pink colonies) were selected for further purification on Columbia Agar with 5% sheep blood (CSB agar, Biorad, Lokeren, Belgium) at 37 °C for 16–24 h. Identification was performed using MALDI-TOF mass spectrometry, with a score value ≥ 2.0 considered as reliable species identification, referenced to the MBT Compass Library (version 2023).

#### 4.2.3. Selective Isolation of Resistant Strains

In addition to the standard isolation procedures, selective pressure screening was performed in parallel. For *E. coli*, MacConkey agar plates (Thermo Scientific™ Oxoid™, Basingstoke, UK) supplemented with 1 mg/L cefotaxime (Merck Life Science, Hoeilaart, Belgium) (incubated at 37 °C for 18–22 h) were used to isolate third-generation cephalosporin-resistant strains. For enterococci, Slanetz–Bartley agar plates (Thermo Scientific™ Oxoid™, Basingstoke, UK) supplemented with 4 mg/L linezolid (Selleckchem, Houston, TX, USA) were employed to detect linezolid-resistant *E. faecalis* and *E. faecium*. All isolates from selective media underwent the same purification and identification procedures as described above.

#### 4.2.4. *Campylobacter* spp. Isolation

BPW homogenous solution was incubated for 4–6 h at 37 °C. After the initial incubation, the enriched solution was transferred to a microaerophilic environment (6.5% O_2_, 5.5%CO_2_) and further incubated at 42 °C for 48 h. Subsequently, 100 µL of the enriched solution was streaked onto modified Charcoal Cefoperazone Deoxycholate Agar (m-CCDA, Thermo Scientific, Waltham, MA, USA) and Campyfood Agar (CFA, bioMérieux, Marcy-l’Étoile, France), using a sterile cell spreader and incubated under microaerophilic conditions at 42 °C for 40–48 h. Colonies exhibiting typical *Campylobacter* morphology (small, grey, flat or slightly raised colonies [50]) were selected for further purification on CSB agar (Biorad, Lokeren, Belgium) at 42 °C for 44–48 h under microaerophilic conditions. Identification was performed using MALDI-TOF mass spectrometry, using the MBT Compass Library (version 2023), with biochemical confirmation (oxidase test and hippurate hydrolysis) performed when MALDI-TOF scores were between 1.7 and 2.0. All bacterial isolates were temporarily stored at 2–8 °C on their plate for subsequent antimicrobial susceptibility testing.

### 4.3. Antimicrobial Susceptibility

From each sample, one bacterial isolate from each bacterial species and from each standard and selective isolation was randomly selected to conduct antimicrobial susceptibility testing.

The antimicrobial susceptibility testing of these isolated strains was conducted using the broth microdilution method in agreement with the reference method ISO 20776-1:2019 [51]. For quality control purposes, reference strains were included in each test run: *E. coli* ATCC 25922 for *E. coli* isolates, *E. faecalis* ATCC 29212 for both *E. faecalis* and *E. faecium* isolates, and *C. jejuni* ATCC 33560 for *Campylobacter* spp. isolates.

The isolated *E. coli* strains were tested against amikacin, ampicillin, azithromycin, cefotaxime, ceftazidime, chloramphenicol, ciprofloxacin, colistin, gentamicin, meropenem, nalidixic acid, sulfamethoxazole, tetracycline, trimethoprim, and tigecycline using EUVSEC 3 Sensititre^TM^ plates (Trek Diagnostic Systems; Thermo Scientific, Waltham, MA, USA) antimicrobial panels according to the manufacturer’s guidelines.

The isolated *E. faecalis* and *E. faecium* strains were tested against ampicillin, chloramphenicol, ciprofloxacin, daptomycin, erythromycin, gentamicin, linezolid, quinupristin/dalfopristin, teicoplanin, tetracycline, tigecycline, and vancomycin using EUVENC Sensititre^TM^ plates (Trek Diagnostic Systems; Thermo Scientific, Waltham, MA, USA). For enterococci strains grown on media supplemented with linezolid, MIC_24h_ was compared with measures after 24 h of additional incubation (MIC_48h_) to assess whether any differences would be observed for linezolid and chloramphenicol, taking into account the potential inducibility of these resistances [45].

The isolated *Campylobacter jejuni* and *Campylobacter coli* strains were tested against chloramphenicol, ciprofloxacin, erythromycin, gentamicin, tetracycline, and ertapenem using EUCAMP3 Sensititre^TM^ plates (Trek Diagnostic Systems; Thermo Scientific, Waltham, MA, USA).

The epidemiological cut-off values (ECOFFs) used for the interpretation as either wild-type (WT) or non-wild-type (NWT) isolates followed the EFSA manual for reporting AMR data [2] and are summarized in Supplementary Materials (Supplementary Material Table S1). Isolates with an MIC strictly higher than the EUCAST ECOFFs [2] were referred to as NWT isolates or as isolates with phenotypically detectable acquired resistance.

### 4.4. Data Analysis

#### 4.4.1. Statistical Methods

Descriptive statistics were calculated to summarize the sample characteristics, including production location, animal species, and meat type (e.g., muscle tissue, bones, fat, organs, and skin and hair). For mixed animal species and mixed meat types, including the partial or whole body, samples were labelled as mix/mixed in the descriptive statistics. The prevalence of *Escherichia coli*, *Enterococcus faecalis*, *Enterococcus faecium*, and *Campylobacter* spp. in the sampled raw-meat products (RMBDs) was described, along with the frequency distributions of categorical variables. Data were entered into Microsoft Excel (version 16.98).

#### 4.4.2. Risk Factors

The association between potential risk factors (production location, meat components, and animal sources) and bacterial presence and MDR detection) was analyzed by means of logistic regression. Separate models were developed for each bacterial species (*E. coli*, *E. faecalis*, *E. faecium*, and *Campylobacter* spp.) and MDR, as long as there were sufficient positive samples. The dependent variable in each model was the presence or absence of the specific bacterial species or the detection of MDR bacteria. First, univariate analysis was conducted to examine associations between potential risk factors and bacterial presence or the detection of MDR indicator isolates. Considering the sample size (N = 50) and the type of variables (categorical variables), the univariate analysis used was Fisher’s exact test. For the multivariate analysis, variables with a *p* < 0.2 in the univariate analysis were incorporated into the binary logistic regression models. Production location was forced into all models to correct for potential confounding. Model selection employed an ENTER approach. The Hosmer–Lemeshow test was used to assess model fit, and the area under the receiver operating characteristic (ROC) curve was calculated to evaluate model discrimination. All results were expressed as odds ratios (OR) with 95% confidence intervals (CI), with statistical significance set at *p* < 0.05.

All data analysis was performed in IBM SPSS Statistics (version 30.0, IBM Corporation, Armonk, NY, USA).

## 5. Conclusions

This study revealed widespread bacterial contamination in commercial raw-meat-based diets for pets in Belgium, with 90% of samples containing *E. coli*. Notably, multidrug-resistant isolates were detected in 30% of the RMBDs, and isolates exhibiting last-resort antimicrobial resistance were observed, including one *E. faecalis* strain with acquired resistance to vancomycin and daptomycin. These findings highlight the need for improved production standards and enhanced regulatory oversight in the raw-pet-food industry and the need to raise awareness in pet owners.

## Figures and Tables

**Table 1:** Distribution of MIC values of 45 strains of *E. coli* isolated from RMBDs.

Antimicrobial Agent	Number of Strains with MIC (mg/L) and Tested Range																Wild-Type		Non-Wild-Type	
	0.015	0.03	0.06	0.125	0.25	0.5	1	2	4	8	16	32	64	128	256	512	[*n*]	[%]	[*n*]	[%]
Amikacin									45	0	0	0	0	0			45	100.0%	0	0.0%
Gentamicin						37	7	0	0	0	1						44	97.8%	1	2.2%
Ampicillin							2	10	14	2	0	17					28	62.2%	17	37.8%
Azithromycin								4	19	22	0	0	0				45	100.0%	0	0.0%
Cefotaxime					34	0	1	2	8								34	75.6%	11	24.4%
Ceftazidime					34	0	2	1	2	6							34	75.6%	11	24.4%
Chloramphenicol										42	0	0	3				42	93.3%	3	6.7%
Ciprofloxacin	36	2	0	4	2	0	0	0	0	1							38	84.4%	7	15.6%
Nalidixic Acid									39	1	0	1	4				40	88.9%	5	11.1%
Colistin							43	0	0	0	2						43	95.6%	2	4.4%
Meropenem		44	0	1	0	0	0	0	0	0	0						45	100.0%	0	0.0%
Sulfamethoxazole										15	15	2	1	0	0	12	33	73.3%	12	26.7%
Trimethoprim					17	21	0	0	0	0	7						38	84.4%	7	15.6%
Tetracycline								31	0	0	0	14					31	68.9%	14	31.1%
Tigecycline					44	1	0	0	0	0							45	100.0%	0	0.0%

The **|** in the table represent ECOFFs [2] used to interpret MICs of *E. coli*; the grey-shadow background in the table above represents the test ranges of the antibiotics; the leftmost cell of each grey-background row means that it is less than or equal to the corresponding tested value, and the rightmost cell of each grey-background row means that it is greater than the corresponding tested value.

**Table 2:** Distribution of MIC values of 31 strains of *E. faecalis* isolated from RMBDs.

Antimicrobial Agent	Number of Strains with MIC (mg/L) and Tested Range													Wild-Type		Non-Wild-Type	
	0.03	0.06	0.125	0.25	0.5	1	2	4	8	16	32	64	128	[*n*]	[%]	[*n*]	[%]
Ampicillin					5	15	9	1	0	0	0	1		30	96.8%	1	3.2%
Chloramphenicol								5	24	2	0	0	0	31	100.0%	0	0.0%
Ciprofloxacin			0	0	3	22	3	3	0	0				31	100.0%	0	0.0%
Daptomycin				0	0	21	6	3	1	0	0			30	96.8%	1	3.2%
Erythromycin						12	11	0	1	1	0	0	6	23	74.2%	8	25.8%
Gentamicin									19	12	0	0	0	31	100.0%	0	0.0%
Linezolid					0	4	26	1	0	0	0	0		31	100.0%	0	0.0%
Quinupristin/Dalfopristin					1	2	0	1	14	13	0	0		3	9.7%	28	90.3%
Teicoplanin					31	0	0	0	0	0	0	0		31	100.0%	0	0.0%
Vancomycin						17	7	6	1	0	0	0	0	30	96.8%	1	3.2%
Tetracycline						15	0	0	0	0	2	9	5	15	48.4%	16	51.6%
Tigecycline	5	13	12	1	0	0	0	0						31	100.0%	0	0.0%

The **|** in the table represent ECOFFs [2] used to interpret MICs of *E. faecalis*. *E. faecalis* is intrinsically resistant to quinupristin/dalfopristin. The grey-shadow background in the table represents the test ranges of the antibiotics; the leftmost cell of each grey-background row means that it is less than or equal to the corresponding tested value, and the rightmost cell of each grey-background row means that it is greater than the corresponding tested value. The range of gentamicin extended up to 1024 mg/L, but no values were observed above 128 mg/L.

**Table 3:** Distribution of MIC values of 23 strains of *E. faecium* isolated from RMBDs.

Antimicrobial Agent	Number of Strains with MIC (mg/L) and Tested Range													Wild-Type		Non-Wild-Type	
	0.03	0.06	0.125	0.25	0.5	1	2	4	8	16	32	64	128	[*n*]	[%]	[*n*]	[%]
Ampicillin					2	8	10	1	1	0	0	1		21	91.3%	2	8.7%
Chloramphenicol								7	14	0	1	1	0	22	95.7%	1	4.3%
Ciprofloxacin			0	1	3	4	6	7	2	0				21	91.3%	2	8.7%
Daptomycin				0	1	1	9	12	0	0	0			23	100.0%	0	0.0%
Erythromycin						12	6	2	1	0	0	0	2	20	87.0%	3	13.0%
Gentamicin									21	2	0	0	0	23	100.0%	0	0.0%
Linezolid					0	0	21	1	1	0	0	0		22	95.7%	1	4.3%
Quinupristin/Dalfopristin					5	4	3	10	1	0	0	0		9	39.1%	14	60.9%
Teicoplanin					22	1	0	0	0	0	0	0		23	100.0%	0	0.0%
Vancomycin						18	3	2	0	0	0	0	0	23	100.0%	0	0.0%
Tetracycline						16	0	0	0	0	1	2	4	16	69.6%	7	30.4%
Tigecycline	3	8	10	2	0	0	0	0						23	100.0%	0	0.0%

The **|** in the table represent ECOFFs [2] used to interpret MICs of *E. faecium*. The grey-shadow background in the table represents the test ranges of the antibiotics; the leftmost cell of each grey-background row means that it is less than or equal to the corresponding tested value, and the rightmost cell of each grey-background row means that it is greater than the corresponding tested value. The range of gentamicin extended up to 1024 mg/L, but no values were observed above 128 mg/L.

**Table 4:** Resistance Profiles of 17 acquired MDR strains isolated on non-selective medium.

Isolates	Antibiotic Resistance Profiles	No. of Antibiotic (Sub)-Class Resistances
*E. coli*		
EC01	CTX-CAZ-CIP-NAL-COL	3
EC02	AMP-CHL-SXT-TET-TMP	5
EC03	AMP-CTX-CAZ-CHL-CIP-NAL-TET-TMP	6
EC04	AMP-SXT-TET-TMP	4
EC05	AMP-CTX-CAZ-SXT-TET	4
EC06	AMP-CTX-CAZ-SXT-TET	4
EC07	AMP-CTX-SXT	3
EC08	AMP-SXT-TMP	3
EC09	GEN-AMP-CTX-CAZ-CIP-SXT-TET-TMP	7
EC10	AMP-CTX-CAZ-TET	3
EC11	AMP-CTX-CAZ-TET	3
EC12	AMP-CTX-CAZ-CHL-SXT-TET-TMP	6
EC13	AMP-CIP-NAL-SXT-TET-TMP	5
EC14	CAZ-CIP-NAL-COL-SXT	4
*E. faecalis*		
EFS1	AMP-ERY-QD-TET	3 *
*E. faecium*		
EFM1	CHL-ERY-LZD-QD-TET	5
EFM2	AMP-CIP-ERY-QD-TET	5

* *E. faecalis* has the intrinsic resistance to quinupristin/dalfopristin, not taken into account in the number of antibiotic class resistances. AMP: Ampicillin, CAZ: Ceftazidime, CHL: Chloramphenicol, CIP: Ciprofloxacin, COL: Colistin, CTX: Cefotaxime, ERY: Erythromycin, GEN: Gentamicin, LZD: Linezolid, NAL: Nalidixic Acid, QD: Quinupristin/Dalfopristin, SXT: Sulfamethoxazole, TET: Tetracycline, TMP: Trimethoprim. The classification of antibiotics is shown in Supplemental Material Table S2.

**Table 5:** Contingency table of categorical univariable.

			*E. coli* (N = 45)				*E. faecalis* (N = 31)				*E. faecium* (N = 23)				Samples with MDR Indicator Strains (N = 15)			
Variable		[N]	Present [*n*/N]	[%]	OR (95% CI)	*p*-Val	Present [*n*/N]	[%]	OR (95% CI)	*p*-Val	Present [*n*/N]	[%]	OR (95% CI)	*p*-Val	Detected [*n*/N]	[%]	OR (95% CI)	*p*-Val
Production Location	Belgium	17	17/17	100.0	N/A	0.279	8/17	47.1	0.42 (0.12–1.45)	0.216	13/17	76.5	11.9 (2.83–50.25)	<0.001	6/17	35.3	1.36 (0.38–4.95)	0.744
	Netherlands	28	25/28	89.3	1.00 (ref)	-	19/28	67.9	1.00 (ref)	-	6/28	21.4	1.00 (ref)		8/28	28.6	1.00 (ref)	
	Others	5	3/5	60	0.18 (0.02–1.55)	0.155	4/5	80.0	1.90 (0.18–19.48)	1.000	4/5	80	14.67 (1.37–156.89)	0.021	1/5	20	0.63 (0.60–6.49)	1.000
	Overall test				0.032				0.320				<0.001				0.905	
Muscle tissue	Present	33	29/33	87.9	0.45 (0.05–4.01)	0.650	18/33	54.6	0.37 (0.10–1.37)	0.218	15/33	45.5	0.94 (0.29–3.03)	1.000	12/33	36.4	2.68 (0.64–11.12)	0.209
	Absent	17	16/17	94.1			13/17	76.5			8/17	47.1			3/17	17.6		
Offal	Present	22	21/22	95.5	3.50 (0.36–33.82)	0.368	14/22	63.6	1.13 (0.35–3.59)	1.000	8/22	36.4	0.51 (0.16–1.55)	0.264	5/22	22.7	0.53 (0.15–1.87)	0.367
	Absent	28	24/28	85.7			17/28	60.7			15/28	53.6			10/28	35.7		
Bones	Present	16	13/16	81.3	0.27 (0.04–1.81)	0.311	11/16	68.8	1.54 (0.44–5.42)	0.549	6/16	37.5	0.60 (0.18–2.02)	0.546	4/16	25.0	0.70 (0.18–2.66)	0.746
	Absent	34	32/34	94.1			20/34	58.8			17/34	50			11/34	32.4		
Fat	Present	6	6/6	100	N/A	1.000	4/6	66.7	1.26 (0.21–7.64)	1.000	1/6	16.7	0.20 (0.22–1.854)	0.199	1/6	16.7	0.43 (0.05–4.02)	0.654
	Absent	44	39/44	88.6			27/44	61.4			22/44	50			14/44	31.8		
Skin and Hair	Present	6	6/6	100	N/A	1.000	4/6	66.7	1.26 (0.21–7.64)	1.000	0/6	0	N/A	0.025	1/6	16.7	0.43 (0.05–4.02)	0.654
	Absent	44	39/44	88.6			27/44	61.4			23/44	52.3			14/44	31.8		
Conventional Livestock	Present	36	33/36	91.7	1.83 (0.27–12.38)	0.611	20/36	55.6	0.34 (0.08–1.43)	0.118	17/36	47.2	1.19 (0.34–4.14)	0.517	9/36	25.0	0.44 (0.12–1.63)	0.185
	Absent	14	12/14	85.7			11/14	78.6			6/14	42.9			6/14	42.9		
Minor Livestock	Present	14	12/14	85.7%	0.55 (0.08–3.67)	0.611	10/14	71.4	1.79 (0.47–6.79)	0.522	7/14	50.0	1.25 (0.36–4.31)	0.761	8/14	57.1	5.52 (1.43–21.14)	0.016
	Absent	36	33/36	91.7%			21/36	58.3			16/36	44.4			7/36	19.4		
Wild Animals	Present	4	4/4	100	N/A	0.647	1/4	25.0	0.18 (0.02–1.85)	0.147	3/4	75.0	3.90 (0.38–40.37)	0.246	2/4	50.0	2.54 (0.32–19.96)	0.346
	Absent	46	41/46	89.1			30/46	65.2			20/46	43.5			13/46	28.3		

N/A: Not available; the odds ratio (OR) could not be calculated precisely or tends toward infinity due to the presence of zero cell(s) in the contingency table.

## Data Availability

The raw data supporting the conclusions of this article will be made available by the authors on request.

## Supplementary Materials

The following supporting information can be downloaded at: https://www.mdpi.com/article/10.3390/antibiotics15030282/s1. Supplementary Material Table S1: Antimicrobial categories considered for MDR classes and ECOFFs (EFSA, 2025) for *E. coli*, *E. faecalis*, *E. faecium*, *C. jejuni* and *C. coli*. Supplementary Material Table S2: The distribution of MIC values of 17 strains of *E. coli* isolated from selective agar plates supplemented with 1 mg/L cefotaxime from RMBDs. Supplementary Material Table S3: The MIC_24h/48h_ values of *E. faecium* isolated from selective agar plates supplemented with 4 mg/L linezolid from RMBDs.
