# Supplementary material for: Occurrence of Antimicrobial Resistance in Indicator Bacteria and Campylobacter spp. Isolated from Commercial Raw-Meat-Based Food for Dogs and Cats in Belgium
Source: Antibiotics (Basel). 2026 Mar 10;15(3):282. doi: 10.3390/antibiotics15030282 (PMC13024599; doi:10.3390/antibiotics15030282)
Supplement: Supplementary file 1 [file antibiotics-15-00282-s001.zip › Supplementary material Table S1_ AMR categories and ECOFFs_20260130.pdf]

Supplementary Material Table S1. Antimicrobial categories considered for MDR classes and ECOFFs [2] for *E. coli*, *E. faecalis*, *E. faecium*, *C. jejuni* and *C. coli*.

Antibiotic (sub-)classes	Antibiotic (abbr.)	*E. coli* ECOFF(µg/ml)
Aminoglycosides	Amikacin (AMK)	8
Aminoglycosides	Gentamicin (GEN)	2
Penicillins	Ampicillin (AMP)	8
Macrolides	Azithromycin (AZM)	16
3rd-gen Cephalosporins	Cefotaxime (CTX)	0.25
3rd-gen Cephalosporins	Ceftazidime (CAZ)	0.5
Carbapenems	Imipenem (IMI)	0.5
Carbapenems	Ertapenem (ETP)	0.06
Carbapenems	Meropenem (MEM)	0.125
(Fluoro)quinolones	Ciprofloxacin (CIP)	0.06
Polymyxins	Colistin (COL)	2
Quinolones	Nalidixic Acid (NAL)	8
Sulfonamides	Sulfamethoxazole (SXT)	64
Tetracyclines	Tetracycline (TET)	8
Diaminopyrimidines	Trimethoprim (TMP)	2
Glycylcyclines	Tigecycline (TGC)	0.5
Cephamycins	Cefoxitin (FOX)	8
β-lactam/β-lactamase inhibitor combinations	Cefotaxime/clavulanic acid (CTX-CLA)	0.25
β-lactam/β-lactamase inhibitor combinations	Ceftazidime/clavulanic acid (CAZ-CLA)	0.5
Amphenicols	Chloramphenicol (CHL)	16

Antibiotic (sub-)classes	Antibiotic (abbr.)	*E. faecalis* ECOFF(µg/ml)	*E. faecium* ECOFF(µg/ml)
β-Lactams	Ampicillin (AMP)	4	4
Amphenicols	Chloramphenicol (CHL)	32	32
Fluoroquinolones	Ciprofloxacin (CIP)	4	4
Lipopeptides	Daptomycin (DAP)	4	8
Macrolides	Erythromycin (ERY)	4	4
Aminoglycosides	Gentamicin (GEN)	64	32
Oxazolidinones	Linezolid (LZD)	4	4
Streptogramins	Quinupristin/Dalfopristin (QD)	1 (intrinsic resistance)	1
Glycopeptides	Teicoplanin (TEI)	2	2
Tetracyclines	Tetracycline (TET)	4	4
Glycylcyclines	Tigecycline (TGC)	0.25	0.25
Glycopeptides	Vancomycin (VAN)	4	4

Antibiotic (sub-)classes	Antibiotic (abbr.)	*C. jejuni* ECOFF (µg/ml)	*C. coli* ECOFF (µg/ml)
Amphenicols	Chloramphenicol (CHL)	16	16
Fluoroquinolones	Ciprofloxacin (CIP)	0.5	0.5
Macrolides	Erythromycin (ERY)	4	8
Aminoglycosides	Gentamicin (GEN)	2	2
Tetracyclines	Tetracycline (TET)	1	2
Carbapenems	Ertapenem (ETP)	0.5	0.5

Reference:

[2] EFSA (European Food Safety Authority), Amore G, Beloeil P- A, Garcia Fierro R, Guerra B, Rizzi V and Stoicescu A- V, 2025. Manual for reporting 2024 antimicrobial resistance data under Directive 2003/99/EC and Commission Implementing Decision (EU) 2020/1729. *EFSA supporting publication* 2025: 22(1):EN-9238. 39 pp. doi:10.2903/sp.efsa.2025.EN-9238
