# Supplementary material for: Occurrence of Antimicrobial Resistance in Indicator Bacteria and Campylobacter spp. Isolated from Commercial Raw-Meat-Based Food for Dogs and Cats in Belgium
Source: Antibiotics (Basel). 2026 Mar 10;15(3):282. doi: 10.3390/antibiotics15030282 (PMC13024599; doi:10.3390/antibiotics15030282)
Supplement: Supplementary file 1 [file antibiotics-15-00282-s001.zip › Supplementary material Table S2_E coli CTX_20260130.pdf]

Supplementary Material Table S2. The distribution of MIC values of 17 strains of *E. coli* isolated from selective agar plates supplemented with 1 mg/L cefotaxime from RMBDs

Antimicrobial Agent	Number of Strains with MIC (mg/L) and tested range																Wild Type		Non-Wild Type	
	0.015	0.03	0.06	0.125	0.25	0.5	1	2	4	8	16	32	64	128	256	512	[n]	[%]	[n]	[%]
Amikacin									17	0	0	0	0	0			17	100.0%	0	0.0%
Gentamicin						7	5	1	2	0	2						13	76.5%	4	23.5%
Ampicillin							0	0	0	0	0	17					0	0.0%	17	100.0%
Azithromycin								1	5	11	0	0	0				17	100.0%	0	0.0%
Cefotaxime					0	0	0	3	14								0	0.0%	17	100.0%
Ceftazidime					0	0	0	1	4	12							0	0.0%	17	100.0%
Chloramphenicol										13	0	0	4				13	76.5%	4	23.5%
Ciprofloxacin	6	2	1	3	1	2	0	0	0	2							9	52.9%	8	47.1%
Nalidixic Acid									12	2	0	0	3				14	82.3%	3	17.7%
Colistin							17	0	0	0	0						17	100.0%	0	0.0%
Meropenem		17	0	0	0	0	0	0	0	0	0						17	100.0%	0	0.0%
Sulfamethoxazole										3	3	2	3	0	0	6	11	64.7%	6	35.3%
Trimethoprim					6	5	0	1	0	0	5						12	70.6%	5	29.4%
Tetracycline								6	1	0	0	10					7	41.2%	10	58.8%
Tigecycline					17	0	0	0	0	0							17	100.0%	0	0.0%

The | in the table represent ECOFFs [2] used to interpret MICs of *E. coli*; The grey shadow background in the table represent the test ranges of the antibiotics., and the leftmost cell of each grey background row means that it is less than or equal to the corresponding tested value, the rightmost cell of each gery background row means that it is greater than the corresponding tested value.

Reference:

[2] EFSA (European Food Safety Authority), Amore G, Beloeil P- A, Garcia Fierro R, Guerra B, Rizzi V and Stoicescu A- V, 2025. Manual for reporting 2024 antimicrobial resistance data under Directive 2003/99/EC and Commission Implementing Decision (EU) 2020/1729. *EFSA supporting publication* 2025: 22(1):EN-9238. 39 pp. doi:10.2903/sp.efsa.2025.EN-9238
