# Supplementary material for: Occurrence of Antimicrobial Resistance in Indicator Bacteria and Campylobacter spp. Isolated from Commercial Raw-Meat-Based Food for Dogs and Cats in Belgium
Source: Antibiotics (Basel). 2026 Mar 10;15(3):282. doi: 10.3390/antibiotics15030282 (PMC13024599; doi:10.3390/antibiotics15030282)
Supplement: Supplementary file 1 [file antibiotics-15-00282-s001.zip › Supplementary material Table S3_enterococci_LZD_20260130.pdf]

Supplementary Material Table S3.The MIC$_{24h/48h}$ values of *E. faecium* isolated from selective agar plates supplemented with 4 mg/L linezolid from RMBDs

Antimicrobial Agent	ECOFF(mg/L）	Isolates and MIC$_{24h}$ values (mg/L）/MIC$_{48h}$ values (mg/L)				
		LR-EFM1	LR-EFM2	LR-EFM3	LR-EFM4	LR-EFM5
Ampicillin	4	4	**8**	4	**8**	≤0,5
Chloramphenicol*	32	**64/128**	32/32	**64/128**	32/**64**	32/**64**
Ciprofloxacin	4	4	4	4	1	4
Erythromycin	4	≤1	≤1	**8**	4	≤1
Gentamicin	32	≤8	≤8	≤8	16	≤8
Linezolid*	4	**16/32**	4/**16**	**8/16**	**8/16**	4/**8**
Quinupristin/dalfopristin	1	1	**4**	**4**	**4**	**4**
Teicoplanin	2	≤0,5	≤0,5	≤0,5	≤0,5	≤0,5
Tetracycline	4	≤1	**64**	**64**	**64**	**128**
Vancomycin	4	≤1	4	≤1	≤1	2
Daptomycin	8	4	4	4	4	2
Tigecycline	0.25	0,12	0,12	0,06	0,12	0,25

The stars in the table indicated the two antimicrobials for which an additional reading after 48h of incubation of the broth microdilution plates was added on the right of the corresponding cell in the table. MIC values in bold are strictly higher than the EUCAST ECOFF [2].

Reference:

[2] EFSA (European Food Safety Authority), Amore G, Beloeil P- A, Garcia Fierro R, Guerra B, Rizzi V and Stoicescu A- V, 2025. Manual for reporting 2024 antimicrobial resistance data under Directive 2003/99/EC and Commission Implementing Decision (EU) 2020/1729. *EFSA supporting publication* 2025: 22(1):EN-9238. 39 pp. doi:10.2903/sp.efsa.2025.EN-9238
